# Direct quantification of ecological drift at the population level in synthetic bacterial communities

**DOI:** 10.1038/s41396-020-00754-4

**Published:** 2020-08-27

**Authors:** Stilianos Fodelianakis, Adriana Valenzuela-Cuevas, Alan Barozzi, Daniele Daffonchio

**Affiliations:** 1 King Abdullah University of Science and Technology (KAUST), Biological and Environmental Sciences and Engineering Division (BESE), Red Sea Research Center, Thuwal, 23955-6900 Saudi Arabia; 2 Present Address: Stream Biofilm and Ecosystem Research Laboratory, Ecole Polytechinque Fédérale de Lausanne, Lausanne, Switzerland

**Keywords:** Microbial ecology, Microbial communities

## Abstract

In community ecology, drift refers to random births and deaths in a population. In microbial ecology, drift is estimated indirectly via community snapshots but in this way, it is almost impossible to distinguish the effect of drift from the effect of other ecological processes. Controlled experiments where drift is quantified in isolation from other processes are still missing. Here we isolate and quantify drift in a series of controlled experiments on simplified and tractable bacterial communities. We detect drift arising randomly in the populations within the communities and resulting in a 1.4–2% increase in their growth rate variability on average. We further use our experimental findings to simulate complex microbial communities under various conditions of selection and dispersal. We find that the importance of drift increases under high selection and low dispersal, where it can lead to ~5% of species loss and to ~15% increase in β-diversity. The species extinct by drift are mainly rare, but they become increasingly less rare when selection increases, and dispersal decreases. Our results provide quantitative insights regarding the properties of drift in bacterial communities and suggest that it accounts for a consistent fraction of the observed stochasticity in natural surveys.

## Introduction

The assessment and quantification of community stochasticity, i.e., the degree to which communities form and change in a random and non-predictable way, is a major challenge for ecology and more so for microbial ecology [1, 2]. In the past decades, even the mere existence of stochastic, ecologically neutral, processes was questioned [3]. Nowadays, it is widely recognized that the ecological processes that drive community stochasticity act both in the past and in the present [2]. Processes acting in the past include historical contingency [4] (in the sense that past community states can constrain present communities) and priority effects [5], and processes driving community assembly in the present include selection, dispersal, speciation, and drift [6].

Identifying the contribution of each assembly process to stochasticity is even more challenging because all processes except selection, which depends by definition on species identity and on the environment [7], have a stochastic component. For dispersal, the movement and establishment of species across space actively or passively [8], the stochastic component lies in the fact that passive dispersal (e.g., through water currents in the sea) is, in most cases, independent of species identity [2]. Speciation, i.e., the rise of novel species via the accumulation of genetic variation, is also partly stochastic because the mutation is a stochastic process [9, 10].

Ecological drift, i.e., changes in species population size due to random births and deaths [6], is perhaps the only unambiguously stochastic community assembly process in nature [2]. Just like genetic drift, ecological drift is expected to be more significant with decreasing population size [6] because random demographic events will likely matter more the smaller a population is. Indeed, a recent study in plant communities supported this inverse relationship between population size and the effect of drift and highlighted the role of dispersal in connecting communities and effectively increasing the population sizes of species, thus reducing drift’s weight [11].

However, drift’s contribution to stochasticity is still unclear in bacterial communities [2] where the population sizes are generally large, and the effect of drift is hard to quantify in natural systems where the other ecological processes act simultaneously [2, 6, 12, 13]. With typical bacterial densities ranging from millions of cells per ml of seawater to billions of cells per gram of soil or sediment [14, 15], it has been argued that drift might be most important for the rare taxa in bacterial communities [16]. Moreover, in natural surveys, it is almost impossible to distinguish the effect of drift from the effect of intermediate dispersal and/or relatively weak selection using community snapshots (e.g., via 16S rRNA gene amplicon sequencing) [13].

**Fig. 1:**
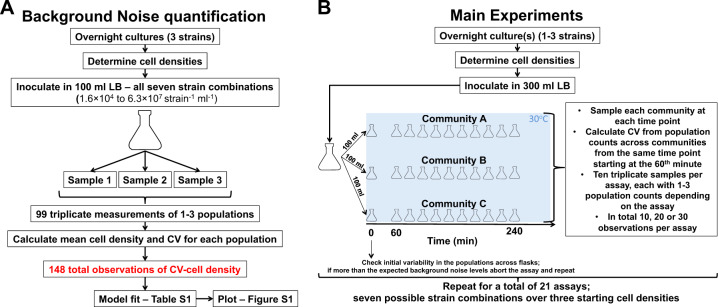
Schematic representation of the experimental assays. **a** Experiments for the quantification of  the “background noise”. **b** Experiments for the quantification of  drift. LB Luria–Bertani broth, CV coefficient of variation. **a** The inoculated cell densities per strain are corresponding to the expected range of cell densities in the main experiments.

**Fig. 2:**
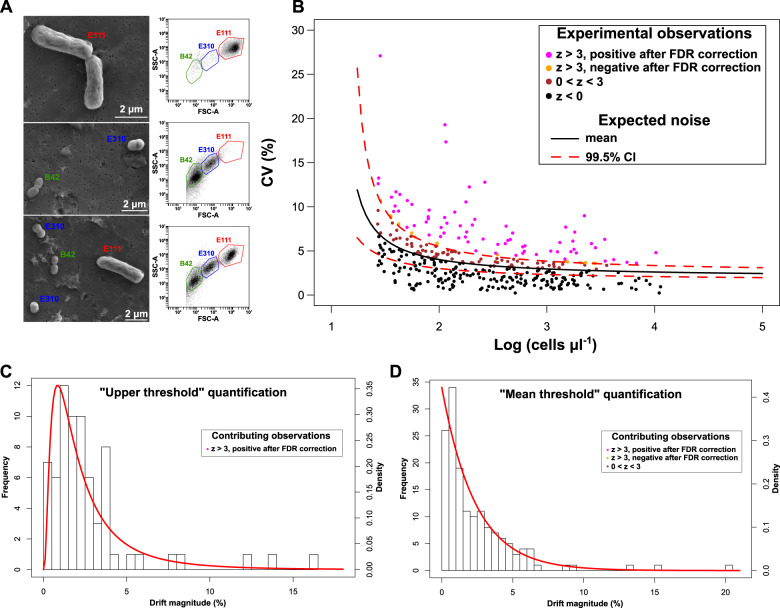
The experimental detection and quantification of drift. **a** Scanning electron microscopy images of example synthetic communities (left) and their respective flow cytometry scatterplots (right). **b** The coefficient of variation (CV, *y* axis, %) among the populations in the three flasks (for a given strain and time point) as a function of the cell density of the respective community (*x* axis, log scale). Curves represent the noise function and its 99.5% confidence intervals, whereas dots represent experimental observations (*n* = 360) that are painted according to their *z*-score from the noise function as per the legend at the upper right. **c** The distribution of the magnitude of drift following quantification based on the “upper threshold”. **d** The distribution of the magnitude of drift following quantification based on the “mean threshold”. **c**, **d** Observations are plotted as a histogram with frequency shown on the left *y* axis and legends indicate the contributing observations with color-coding as per **b**. The density of the fitted distributions (red solid lines - log-normal for **c** and exponential for **d**) is shown on the right *y* axis.

Here, we aimed to detect and quantify drift in isolation from other processes by conducting controlled experiments on simplified synthetic bacterial communities (Fig. 1). We assembled synthetic communities using three strains whose populations we could monitor accurately and in a high temporal resolution using flow cytometry (Fig. 2a). We first quantified the error introduced by the monitoring method and by the experimental handling (referred to as “background noise” or “noise” henceforth—Fig. 1a). We then performed the main experiments by assembling identical triplicate communities of all seven possible combinations, and growing them under identical and controlled conditions starting from three different total cell densities for a total of 21 replicated assays (Fig. 1b). We monitored the bacterial populations using our recently developed method [17] that is based on direct and strain-specific cell detection through flow cytometry. If drift had not occurred, the variation in the population densities across replicate communities at a given time point should be indistinguishable from “noise”. This is because in our experiments replicate communities were originally identical (we verified that before every assay—Fig. 1b) and grew under the same environmental conditions and biotic interactions without dispersal from any external source. Consequently, any deviation from the expected “noise” could only be attributed to random differences in births and deaths, i.e., to ecological drift.

## Materials and methods

### Assembling and monitoring the synthetic bacterial communities

We selected three bacterial strains, a *Chryseobacterium* sp., a *Staphylococcus* sp., and a *Bacillus* sp., from a large collection of soil isolates, and we screened them with fluorescence-independent flow cytometry as we did previously [17]. We measured at an acquisition speed of 14 μl min^−1^ for 2 min per sample and setting a threshold of 10,000 regarding the height signal of the front scatter (FSC-H). Differently than in our previous work, here we acquired all scattering profiles based on growth assays at 30 °C that was the temperature at which we performed all the experiments. In addition, we screened the growing cultures with a temporal resolution of 20, rather than 30, min. We recorded significant interactions among the strains by comparing their single and mixed growth profiles at 30 °C (Dataset 1). These interactions were mainly positive, similarly to what we found previously at other temperatures [17]. We performed all the related growth assays in biological triplicates. All flow cytometry data are available in .fcs format online (http://flowrepository.org) under the “FR-FCM-Z25Q” identifier. Henceforth, when referring to the experiments we use the term “population” to describe the cells of a given strain within a flask at a given assay and the term “community” to describe the total bacterial cells within a flask at a given assay.

### Quantification of “background noise”

Our flow cytometry method for screening the mixed bacterial cultures has an accuracy of 97% for sample densities above 10^5^ cells ml^−1^ [17]. However, at lower densities sampling errors and instrument inconsistencies become increasingly important because the signal-to-noise ratio drops. This can result in substantially different counts among identical samples and can artificially inflate the observed variability. Thus, it was essential to quantify this “noise” before performing the main experiments and subtract it from the observed variability when quantifying drift.

To that end, we made a series of separate experiments to quantify “noise”. In these experiments, we mixed overnight cultures of the three strains in all the seven possible combinations and in final cell densities ranging from 1.6 × 10^4^ to 6.3 × 10^7^ strain^−1^ ml^−1^ (corresponding to the expected range of cell densities in the main experiment, see below), and we measured repeated aliquots from the same flask to determine the coefficient of variation (CV—Fig. 1a). We treated the samples in exactly the same way as in the main experiments to include the effect of sampling errors in our calculations. We acquired in total of 99 triplicate measurements of 1–3 populations for a total of 148 observations (Fig. 1a, Dataset 2). We hypothesized that the level of “noise” should be inversely related to the cell density of the sample, because the signal-to-noise ratio decreases at low cell densities in the flow cytometer. Accordingly, we fit different functions for the dependency of CV to cell density (Supplementary Table S1). Finally, we calculated the 99.5% confidence intervals of the best-fitting function (i.e., Michaelis-Menten) using the *confint* function of the MASS package [18] in R [19], and we defined the false discovery rate based on the number of observations that were above the upper 99.5% confidence interval (Supplementary Fig. S1). Finally, we verified that the levels of noise determined in this study are similar to the variability recorded from technical replicate samples taken during our previous experiment where we used the same bacterial system and instrument with identical settings [17] (Supplementary Fig. S2).

### Main experiments

To quantify drift, we monitored the changes in population densities across identical starting communities incubated under the same environmental conditions (Fig. 1b). To that end, we mixed the three strains in all seven possible combinations, i.e., three monocultures, three mixed cultures of two strains and a mixed culture of all three strains, and in three different starting total cell densities (5 × 10^4^ cells ml^−1^, 10^5^ cells ml^−1^, and 10^6^ cells ml^−1^). To perform each growth assay, we first inoculated the respective strains from overnight pure cultures in a single flask containing 300 ml of Luria–Bertani medium (Sigma). To reach the desired starting total cell density, we estimated the cell density of the overnight pure cultures with flow cytometry [17] and we inoculated the respective volume. We then mixed the culture thoroughly and we immediately split the volume equally into three flasks. We next sampled 500 μl from each flask and we compared the variability in the bacterial populations across the three flasks to the expected “background noise” for the same cell density. In specific, we examined the CVs of the bacterial populations and their *z*-scores compared to the “background noise”, i.e., how many standard deviations an observed CV differs from the expected “background noise” CV at a given cell density. If the observed *z*-scores were larger than 2 (95% CI), we aborted the given experiment because it indicated that we introduced variability when we mixed and split the cultures and thus the starting cultures could not be considered identical; this happened in ~50% of the cases. If the observed *z*-scores were lower than 2, indicating that the recorded variability was not statistically different or was less than the expected variability based on the “background noise”, we proceeded with the experiment, incubating the three flasks in the same chamber (New Brunswick Innova 42R, Eppendorf) at 30 °C and with shaking at 80 rpm.

We recorded the starting densities (Dataset 3, *z*-scores between −6.68 and 1.17) and the densities every 20 min until the end of the fourth hour of incubation starting from the 60th minute. To detect and quantify drift, our main assumption was that any larger-than-expected deviations in the population densities of identical starting communities incubated under the same environmental conditions could only be because of drift. Thus, we compared the observed CVs to the expected CVs based on the “background noise” by deploying the *z*-score. We quantified drift using two different thresholds:

1. The “upper threshold” that focused on excluding false-positive observations. In this quantification, we used a cutoff significance level of *z* > 3 (99.5% CI) and we ignored the lowest 17.57% of positive observations (i.e., 15 observations, corresponding to the FDR level of the “background noise”) to minimize the detection of false positives.
2. The “mean threshold” that focused on excluding false negatives and increasing detectability. In this quantification, we used a cutoff significance level of *z* > 0, meaning that we scored any observation greater than the mean noise function as positive.

The “upper threshold” quantification probably overestimates drift by taking into account only the highest among the recorded CV values while the “mean threshold” quantification underestimates drift by taking into account some low CV values that are very close to the noise levels. Thus, the “upper threshold” and “mean threshold” quantifications do not represent the true levels of drift (which are hard to define whatsoever in the presence of noise) but they rather represent the upper and lower boundaries within which the true levels of drift lie.

### Estimating potential growth variability due to temperature differences within the incubation chamber

To ensure that the recorded variability in the population counts was not due to slight differences in temperature within the incubation chamber, we estimated the potential variability that could have resulted if each strain grew within the extremes of the recorded temperatures in the chamber. For that, we first measured the temperature within each flask at each experimental time point, five times per flask, using a digital immersion thermometer with an accuracy of 0.1 °C. The temperature varied by 0.15 °C ± 0.08 °C on average and by 0.28 °C at maximum. We then calculated the growth rates of each of the strains under the recorded temperature extremes at each time point by interpolating from previously recorded growth rates [17]. We interpolated both with respect to time and with respect to temperature because the previous data were recorded at intervals of 30 min and 0.5 °C (the latter at a range of 25–42 °C). Finally, for each strain, we calculated how the CV in the hypothetical population densities would increase if the strains were constantly growing within the recorded temperature extremes for the duration of the experiment and if the CV was calculated from three observations (like in the real experiments) of the resulting population density distributions. We note that with this analysis, we probably overestimated the hypothetical increase in population densities because we used growth rates from mixed cultures that were generally higher than those in monocultures because of the positive interactions among the strains (Dataset 1).

### Simulations

To simulate drift in complex bacterial communities, we used in silico communities with diversity and abundance distributions similar to nature [20] where drift acts with magnitude according to our experimental data. A conceptual flowchart of the simulations can be found in Supplementary Information (Supplementary Fig. S3). Each simulation involved a metacommunity of 100 communities that were connected with dispersal and that initially contained 2000 species each.

We simulated dispersal occurring in a unidirectional way within a closed system; individuals from community *n* disperse to the community (*n* + 1) and individuals from community 100 disperse back to community 1. The strength of dispersal equaled to the percentage of individuals that disperse to the respective community and it varied between 2 and 20%. Our aim in modeling dispersal in this way was to create a setting where habitat fragmentation was high and therefore drift’s importance is expected to be more pronounced [21, 22], and where there was no gain or loss of individuals from outside the metacommunity.

We simulated selection as differences in the growth rates among species within a community. The growth rates were distributed normally with a mean of 1 (resembling systems at their carrying capacity) and with a standard deviation between 0.071 and 0.167. Growth rates changed at every generation by being re-drawn from the same distribution in an effort to represent fluctuating habitats where a given species is not always favored or disfavored. Therefore, in our simulations, the standard deviation in the growth rates represents the strength of selection, because the higher it is the bigger are the differences in the growth rates in a community and the changes in the growth of a species from generation to generation. The distribution of the abundances in a community at time zero was log-normal (mean = 4, sd = 1.1) and the distribution of the abundances of a given species across all communities was normal with a standard deviation equal to the strength of selection.

The metacommunity grew for 1000 generations under given dispersal and selection conditions with drift, where drift changed the assigned growth rates at every generation according to a distribution based on the defined threshold from the experimental data (“upper” or “mean” threshold). In parallel, an initially identical metacommunity grew under the same dispersal and selection conditions but without drift, meaning that the assigned growth rates at every generation did not change further. More details and an example on how we modeled changes in growth rates due to drift are presented in the Supplementary Text in Supplementary Information.

For a given generation, we calculated the effect of drift by comparing a given community in the metacommunity that grew under drift to the same community in the metacommunity that grew without drift. In specific, we examined the β-diversity by means of the Bray–Curtis (BC) community similarity and the differences in species richness and in Pielou’s evenness among drift-impacted and drift-free communities, calculating the metacommunity-wise mean and standard deviation on all these properties. Moreover, we kept track of the extinct species at the end of each simulation and we mapped their initial relative abundances, but here we report the metacommunity-wise median because the distribution of the relative abundances is skewed (Supplementary Fig. S4). We ran simulations under 50 different scenarios resulting from five levels of selection strength over ten levels of dispersal rate. To estimate the effect of drift on Bray–Curtis similarity in metacommunities with increasing number of species, we ran the same simulation at the highest selection and lowest dispersal levels, at intermediate selection and dispersal and at the lowest selection and highest dispersal, but we changed the number of species; we ran the simulation three times in metacommunities of 500, 1000, 2000, 4000, 6000, 8000, and 10,000 species. We performed all simulations in R. All code is available on GitHub (https://github.com/sfodel/Drift).

### Reported β-diversity in stochastically assembled communities in nature

To compare our simulation results with the results from environmental surveys regarding the β-diversity in stochastically assembled communities, we searched for related studies using the following two criteria: (1) the study cites the works of Stegen and colleagues [12, 13], where the term “undominated” community assembly is presented formally for microbial ecology, (2) the study reports data on the range of the observed β-diversity in terms of Bray–Curtis dissimilarity (or similarity) in stochastically assembled communities, or this range can be inferred from the data presented in that study.

## Results

### Quantifying the background noise

Performing a series of dedicated experiments (Fig. 1a), we found a significant decreasing relationship between “noise” and the cell density of a sample with a Michaelis-Menten function best-fitting that decrease (Supplementary Table S1). Over 82% of the total “noise” observations (122 out of 148) fell below the upper 99.5% confidence interval (CI) limit of the fitted function (Supplementary Fig. S1), resulting in a FDR of 17.57%. We then set two different thresholds based on the fitted “noise” function to quantify drift: the “upper threshold” and the “mean threshold”. The former uses the upper 99.5% CI of the expected “noise” function and the detected FDR to minimize false positives and the latter uses the mean expected “noise” to minimize false negatives (see “Materials and methods” section for more details).

### Quantifying drift

Following the quantification based on the “upper threshold”, drift was detectable in 19.4% (70 out of 360) of the populations (Fig. 2b). Community-wise, it was detectable in at least one population in 26.7% of the communities (56 out of 210). The magnitude of drift following the “upper threshold” quantification, i.e., the observed CV minus the upper 99.5% confidence interval of the expected noise CV, had a median of 2% and it fitted best a log-normal distribution (mean = 0.625, sd = 0.889, Fig. 2c and Supplementary Table S2).

Following the “mean threshold” quantification, drift was detectable in 42.8% (154 out of 360) of the populations (Fig. 2b). Community-wise, it was detectable in at least one population in 29% of the communities (61 out of 210). The magnitude of drift following the “mean threshold” quantification, i.e., the observed CV minus the mean expected noise CV, had a median of 1.4% and it fitted best an exponential distribution (rate = 0.423, Fig. 2d and Supplementary Table S3).

Additional analyses indicated that the observed variability (which we perceived as drift) was random with respect to the experimental parameters and it could not be due to the slight recorded temperature differences within the incubation chamber. First, the *z*-score did not correlate to the cell density, to the identity of the strain, the species richness of the community or to the starting cell concentration (linear mixed-effects model, *n* = 360, −1.439 < *t* < 1.537, 0.155 < *p* < 0.843—Supplementary Table S4) indicating that the deviance from the expected “noise” was random. Second, the recorded deviance cannot be explained by growth under slightly different temperatures because that would cause a lower (and constant) increase of the observed magnitude of drift with time for all three strains than what was recorded (Supplementary Fig. S5).

### Simulating drift in metacommunities under various dispersal and selection scenarios

**Fig. 3:**
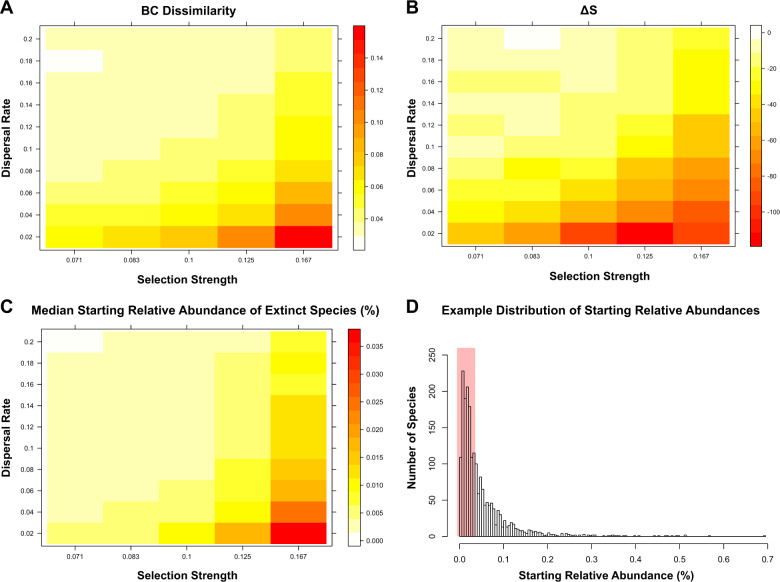
The generated β-diversity, the species loss, and the starting relative abundances of the extinct species due to drift in the simulations under various dispersal and selection scenarios, with drift simulated as per the “upper threshold” quantification. **a** The mean generated β-diversity (Bray–Curtis community dissimilarity − BC) after 1000 generations. **b** The average difference in the number of species between drift-free and drift-impacted communities (Δ*S*) after 1000 generations. Comparisons are made with respect to communities growing without drift such that negative values indicate species loss in drift-impacted communities. **c** The median starting relative abundance (%) of the species that got extinct due to drift after 1000 generations. **d** An example distribution of the average starting relative abundances in the simulated metacommunities, with the red box representing the range of the median relative abundances of extinct species in the different simulations as per **c**.

We next used the experimental results from our simplified bacterial system to simulate drift in complex metacommunities under various scenarios of dispersal and selection. Overall, our simulations showed that drift can generate a substantial amount of β-diversity and can lead to considerable species loss with the extinct species being mostly rare (Fig. 3 and Supplementary Fig. S6). The results were very similar when we used either the “upper threshold” or the “mean threshold” drift distributions (Fig. 2c, d, respectively); β-diversity increases when dispersal is low and selection is high (Fig. 3a and Supplementary Fig. S6a) along with the number of extinct species (Fig. 3b and Supplementary Fig. S6b) that become increasingly less rare (i.e., having higher initial relative abundances on average) under these conditions (Fig. 3c, d and Supplementary Fig. S6c, d). The highest generated β-diversity was 15.1% and 11.7% in terms of BC dissimilarity when using the “upper threshold” and “mean threshold” drift distributions, respectively. The maximum observed species loss due to drift was −112 and −68 species per community on average using the “upper threshold” and “mean threshold” drift distributions, respectively, corresponding to 5.6% and 3.4% of the initial species being lost. The median starting relative abundance of the species that got extinct due to drift was 0.035% and 0.034% at maximum in the simulations using the “upper threshold” and “mean threshold” drift distributions, respectively. The potentially extinct species belong to the “rare” fraction of the communities where most species reside so that the range of potentially extinct species includes 40.4% ± 1.1% of the total species (Fig. 3d and Supplementary Fig. S6d).

**Fig. 4:**
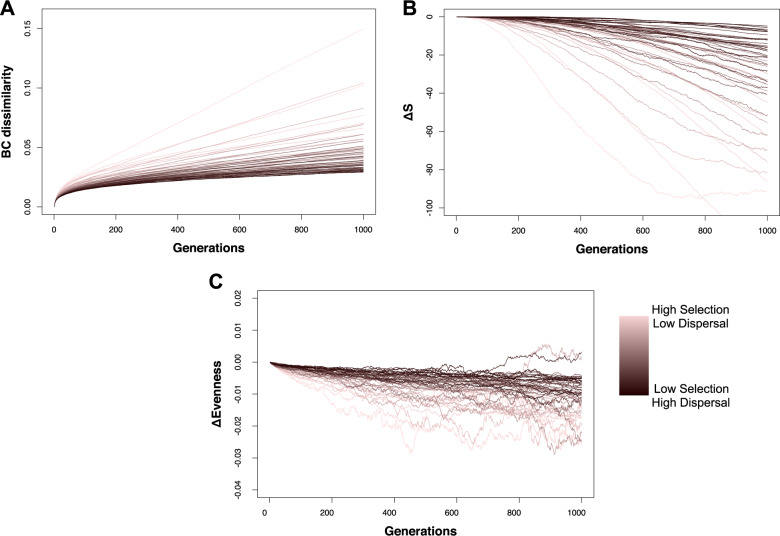
The effect of drift (following the “upper threshold” quantification) on the generated β-diversity, on species loss and on community evenness with increasing time in simulations under various selection and dispersal scenarios. **a** The generated β-diversity (Bray–Curtis dissimilarity). **b** The average difference in the number of species between drift-free and drift-impacted communities (Δ*S*). Negative values indicate species loss in drift-impacted communities. **c** The difference in Pielou’s evenness (ΔEvenness). Negative values indicate lower evenness in drift-impacted communities. The strength of selection and the rate of dispersal change as per the legend on the bottom right.

Given that drift’s effect on species abundances is expected to increase with time [6], we further examined in our simulations the change in the generated β-diversity, species richness, and community evenness due to drift through time. We found that the change in the generated β-diversity saturates after ~500 generations for most selection/dispersal scenarios except at the highest selection and lowest dispersal (Fig. 4a and Supplementary Fig. S7a). On the contrary, the decrease for both species richness and evenness in drift-impacted communities compared to drift-free communities does not saturate for most simulations (Fig. 4b, c and Supplementary Fig. S7b, c), indicating that for these properties the effect of drift would increase with prolonged time.

**Fig. 5:**
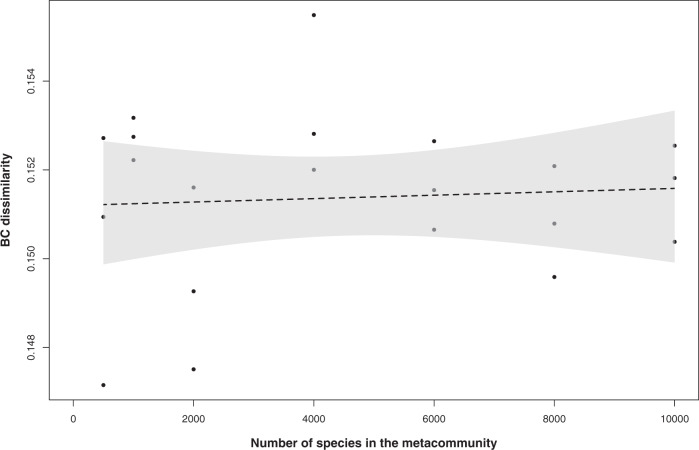
The generated β-diversity by drift (Bray–Curtis dissimilarity − BC), following the “upper threshold” drift quantification, (*y* axis) as a function of the number of species in the metacommunity (*x* axis). Black dots correspond to the values from different simulations (i.e., for metacommunities of 500, 1000, 2000, 4000, 6000, 8000, and 10,000 species, *n* = 3 for each level). The line corresponds to the mean and the shaded gray area to the 99.5% confidence intervals of the fitted, non-significant, linear model (adjusted *R*^2^ = −0.047, *p* = 0.76).

Finally, we hypothesized that the amount of β-diversity generated by drift depends on the diversity of the metacommunity because changes caused by drift would accumulate over an increasing number of species. However, we found that it does not (linear models, adjusted *R*^2^ = −0.047, *p* = 0.76, Fig. 5 for the “upper” and adjusted *R*^2^ = 0.035, *p* = 0.204, Supplementary Fig. S8 for the “mean threshold” quantification), with the generated β-diversity remaining at the same levels even in metacommunities containing 10,000 species under the highest selection and lowest dispersal. The results were unchanged when we repeated the simulations under two more selection/dispersal scenarios (0.071/0.2 and 0.1/0.1—Supplementary Table S5).

## Discussion

### A direct quantification of drift with population-level resolution

Our study contributes to previous works in microbial ecology by providing a quantification of ecological drift at the population level. In our experiments, we have focused on quantifying explicitly and directly the variability due to random births and deaths across populations that is the definition of drift [6], using an experimental setup that isolates drift from other processes and subtracts the experimental and methodological noise (Fig. 1). In contrast, in previous experimental works that laid the foundation on drift in microbial communities “drift” could be resulting from other processes as well. For example, in two relevant experiments on electrolysis cell reactors [5] and on leaf decomposer communities [23], the observed stochasticity that the authors refer to as “drift” could be resulting from priority effects in addition to random births and deaths, from the methodological noise introduced by sequencing or from the uncontrolled spatial variability. On the contrary, here we quantified drift via direct observations at the population level using absolute cell counting (Fig. 2a) in highly controlled and replicable microbiological assays and we accounted for the major possible sources of experimental variability (Fig. 2b and Supplementary Fig. S5). Our estimates of drift, which we found to be independent of the experimental parameters, could be used to predict drift’s effects in laboratory and natural settings when selection and dispersal can be parameterized. However, even though in our study drift was independent of the examined populations and communities, experiments with other bacterial strains that have a wide spectrum of growth rates and phylogenies could further broaden our estimates on drift’s magnitude and on drift’s effects on α- and β-diversity.

### Drift generates β-diversity by leading mainly rare species to extinction

Fed by our experimental data, our simulations support the existing notion that drift affects mainly rare taxa in microbial communities [16]. Expanding that notion, our results also point that the species loss is more prominent at low dispersal rates and in fluctuating environments where drift can affect non-rare taxa as well. For example, half of the extinct species due to drift in the simulation under the lowest dispersal and highest selection were above the 40th percentile, and thus not in the “rare” tail, of the relative abundance distribution (Fig. 3d and Supplementary Fig. S4). Moreover, the species loss and the resulting negative effect of drift on community evenness did not attenuate with time in most of our simulations (Fig. 4b, c and Supplementary Fig. S7b, c), suggesting that drift may partially drive the skewed microbial distributions in nature [20] and that other processes such as dormancy and speciation are important for counterbalancing species loss over long temporal scales [24]. Notably, in this work, we made no assumptions on species equality like, for example, the neutral model of Hubbell [3] that is used widely to explain stochastic patterns in α- and β-diversity.

Even though we aimed to make our simulation as general as possible by avoiding assumptions on neutrality, our simulations do not by any means reproduce the species interactions and the possible environmental settings that can be found in nature, but rather a range of them. For example, our modeled changes in growth rates could be the result of both abiotic and biotic interactions, but biotic interactions could also be weighted by the abundance of other species in a given community. Similarly, our model metacommunity represents a system of low connectivity because a given community therein is connected with only two other communities via dispersal. Changing the form of dispersal, e.g., by randomizing the community order throughout the generations, would most probably change the model output and would be very interesting to examine in future studies that, for example, ask questions about the effect of habitat fragmentation on microbial community stochasticity. In addition, here we used a dispersal gradient of 2–20% of growing cells because in our previous study we found that dispersal of 20% homogenizes the metacommunity [17] and thus we set the lower limit to one-tenth of that. Lower dispersal would definitely result in higher species loss and generated β-diversity but it is questionable if such a degree of isolation really exists in nature [25, 26].

Interestingly, the β-diversity generated by drift in our simulations even in very diverse metacommunities (Fig. 5 and Supplementary Fig. S8) is a considerable part of the reported β-diversity among stochastically assembled communities in nature [27–35] (Table 1). This suggests that drift is a significant driver of the stochasticity observed in nature along with other stochastic processes such as priority effects [5] and historical contingency [2], which might account for the part of the stochasticity not explained by drift. The unexplained component of the observed stochasticity is further amplified by the noise introduced by sequencing-based community screening [36, 37] that is absent in the approach we have deployed. Moreover, some of the studies that we report in Table 1 for comparison did not deploy phylogenetic null modeling. Thus some community pairs therein could be re-classified as being deterministically driven if phylogenetic null modeling was performed, deflating the amount of observed β-diversity in stochastically assembled communities. At the same time, our estimates are necessarily conservative because in our experimental calculations, we aimed to exclude false-positive observations based on the expected noise distribution (Fig. 2b) and this resulted in <50% of detectability for both the applied quantification thresholds.

**Table 1:** The reported β-diversity (Bray–Curtis community dissimilarity, mean or minimum values) in example studies on natural systems where at least a part of microbial community assembly was stochastic.

Study (in alphabetical order)	Stochasticity component	Reported/inferred β-diversity (Bray–Curtis dissimilarity)	Justification	Reference display element within the respective study
Beaton et al. [25]	Dispersal limitation and random processes leading community assembly	>40%	Minimum observed across all samples	Fig. 5
Graham et al. [26]	Undominated community assembly in 0–15% of community pairs	~30%	Minimum observed across all samples	Fig. 2
Langenheder et al. [27]	Drift contribution 55.71% in the whole metacommunity	~45%	Minimum observed across all samples	Supplementary Fig. S1
Mo et al. [28]	Spatial factors, representing neutral processes, governed community assembly	>40%	Minimum observed across all samples	Fig. 3
Xiao et al. [29]	Undominated community assembly in 8–23% of community pairs	~28.5%	Mean observed across all samples + 1 standard deviation	Table 2
Xiong et al. [30]	Stochastic processes dominating the assembly of adult shrimp gut microbiota	~41.5%	Observed centroid in “adult” sample group	Table 2
Yan et al. [31]	Stochastic processes dominating the assembly of adult fish gut microbiota	67–78%	Observed centroids in “adult” sample groups	Table 2
Zhang et al. [32]	Stochastic processes dominating the assembly of microbial communities in bioreactors	41%	Observed centroid in “Group A” sample group	Table 3
Zhou et al. [33]	Stochastic processes dominating the assembly of microbial communities at the intermediate phases after emulsified vegetable oil amendment	60–62%	Observed centroids in “intermediate phase” sample groups	Table 1

### Methodological constraints and future perspectives

The fact that we did not detect drift in all the populations does not mean that drift did not occur in all of them, but rather points to the constraints of the applied method to determine cell densities. Since births and deaths are inherently stochastic [6], there should have always been differences across the populations of a given strain at a given time point in all the assays. In other words, if we had the perfect method to determine cell densities we would always detect drift. However, this is not the case in reality and thus it is necessary to establish the levels of “noise” and subtract it from the observations, resulting in false-negative observations especially when the level of “noise” is high (e.g. at low cell densities in our study—Fig. 2b). Moreover, detectability also depends on the number of samples per observation. Here we used three samples for both “noise” establishment and during the main experiments (Fig. 1) to calculate CV, but increasing that number could have resulted in narrowing down variability and increasing detectability. However, the desired temporal resolution (20 min) was the limiting factor dictating the maximum number of samples that we could handle efficiently between time points because we were measuring in real-time while the cultures were growing. On top of that, the maximum experimental time was also constrained by the flow cytometer, because if we reached further in time the samples would have high cell densities and would require dilution before measuring; that would further inflate the experimental noise. Recent advances in flow cytometry such as acoustic focusing for parallel measurements [38] could lift some of those constraints by allowing fast measurements of samples with high density and with lower noise levels. In addition, experiments such as ours, where relative abundances can be quantified both by sequencing and without sequencing, could be an ideal way to quantify the introduced sequencing noise in synthetic bacterial communities and thus to provide “sequencing noise” thresholds for field studies. The limiting factor in such experiments is the ability to discriminate the different strains that are used, but recent advances in probabilistic algorithms allow for the use of strains with largely overlapping flow cytometry scattering profiles [39].

Despite its limitations, our study experimentally demonstrates the existence, and offers quantitative insights, of drift in bacterial populations. Our simulations place drift as a process that results in significant loss of α-diversity and gain of β-diversity in microbial communities, especially under low dispersal and in fluctuating environments. Overall, our results serve as a starting point for future studies to investigate the effect of drift in more complex microbial systems when methodological constraints are lifted and more precise cell quantification methods become available.

## Supplementary information

Supplementary Material

Dataset 1

Dataset 2

Dataset 3
