# Supplementary material for: Direct quantification of ecological drift at the population level in synthetic bacterial communities
Source: ISME J. 2020 Aug 27;15(1):55–66. doi: 10.1038/s41396-020-00754-4 (PMC7852547; doi:10.1038/s41396-020-00754-4)
Supplement: Supplementary file 1 — Supplementary Material [file 41396_2020_754_MOESM1_ESM.pdf]

Supplementary Information

Direct quantification of ecological drift at the population level in synthetic bacterial communities

Stilianos Fodelianakis[1*§], Adriana Valenzuela-Cuevas[1], Alan Barozzi[1], Daniele Daffonchio[1*]

[1]King Abdullah University of Science and Technology (KAUST), Biological and Environmental Sciences and Engineering Division (BESE), Red Sea Research Center, Thuwal 23955-6900, Saudi Arabia

*Correspondence to: daniele.daffonchio@kaust.edu.sa, stylianos.fodelianakis@epfl.ch

[§]Current address: Stream Biofilm and Ecosystem Research Laboratory, Ecole Polytechinque Fédérale de Lausanne, Lausanne, Switzerland

Supplementary Figures

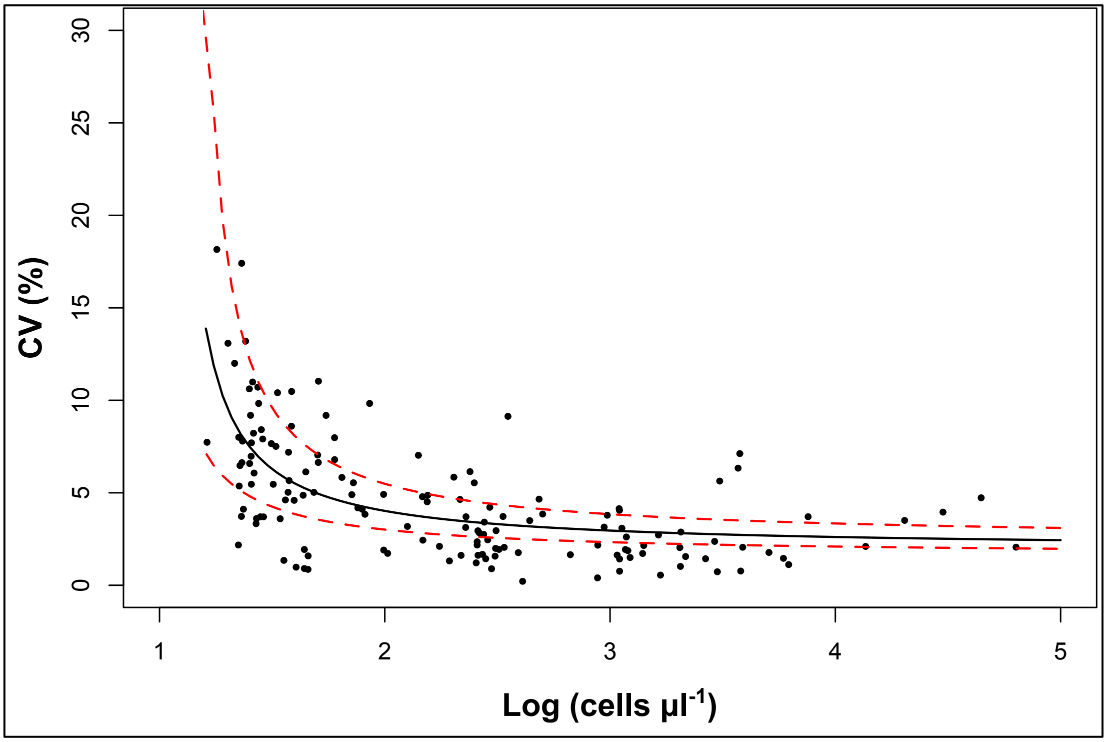

Supplementary Figure S1. The “noise” (y-axis, CV, in percentage) as a function of the cell density of the respective community (x axis, in logarithmic scale). The solid black curve represents the expected “noise” function (Mikaelis-Menten, Supplementary Table S1), and the dashed red curves above and below it represent the upper and lower 99.5% confidence interval functions, respectively.

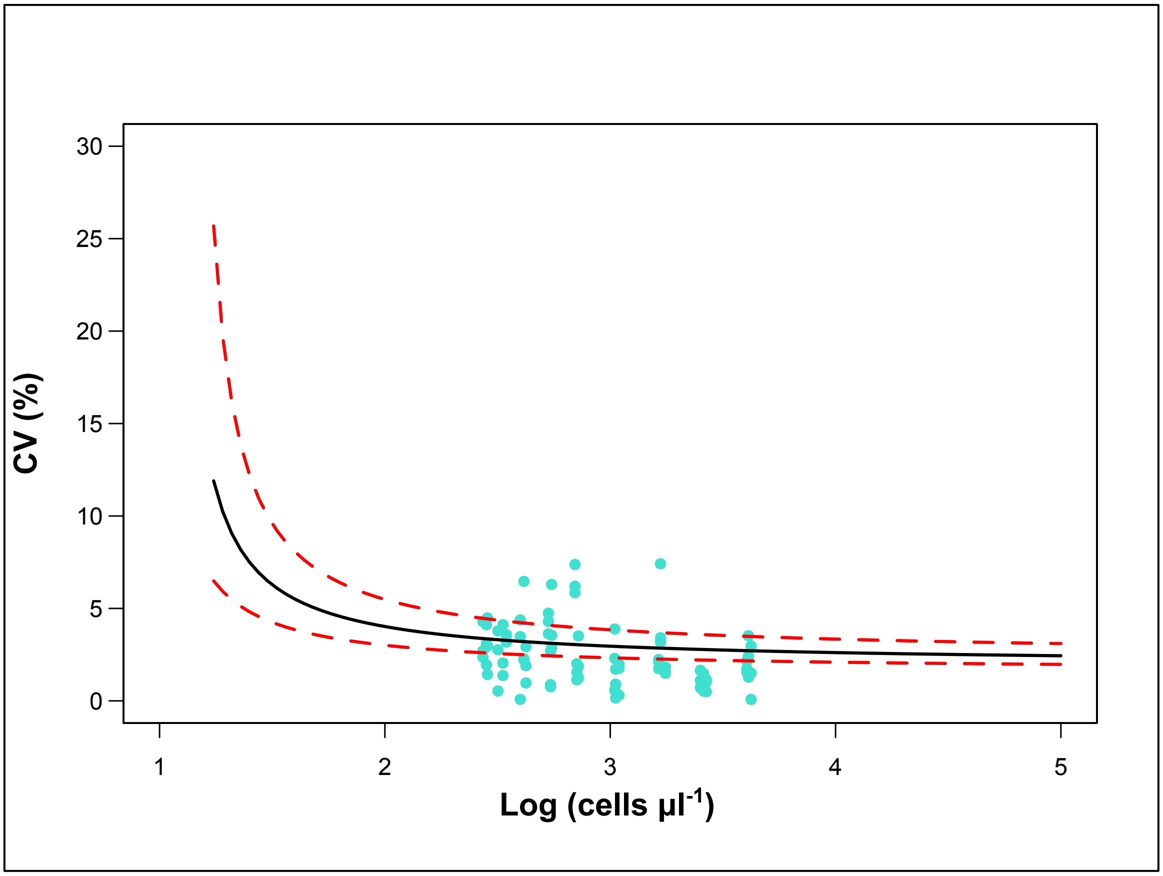

Supplementary Figure S2. The “noise” (y-axis, CV, in percentage) as a function of the cell density of the respective community (x axis, in logarithmic scale) as in Supplementary Figure S2, with cyan dots representing the measured “noise” from technical replicates in a previous experiment where we used the same bacterial system and instrument with identical settings (1). The observations fit to the “noise” distribution of the current experiment with a very similar false discovery rate; 12 out of the 81 observations are above the upper 99.5% confidence interval for a 14.81% rate compared to the 17.57% in the current experiment.

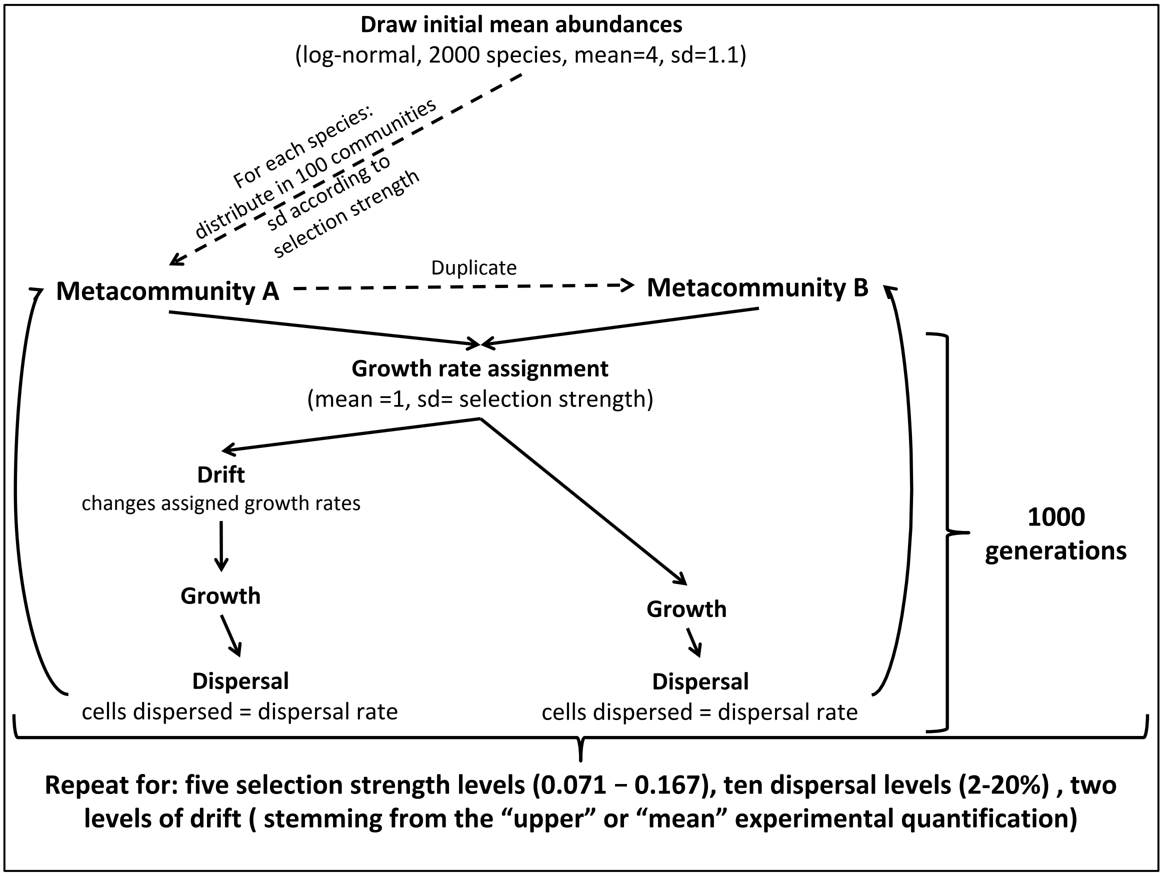

Supplementary Figure S3. Conceptual flowchart of the performed simulations. Solid arrows indicate processes occurring at every generation and dashed arrows indicate processes occurring only before the first generation.

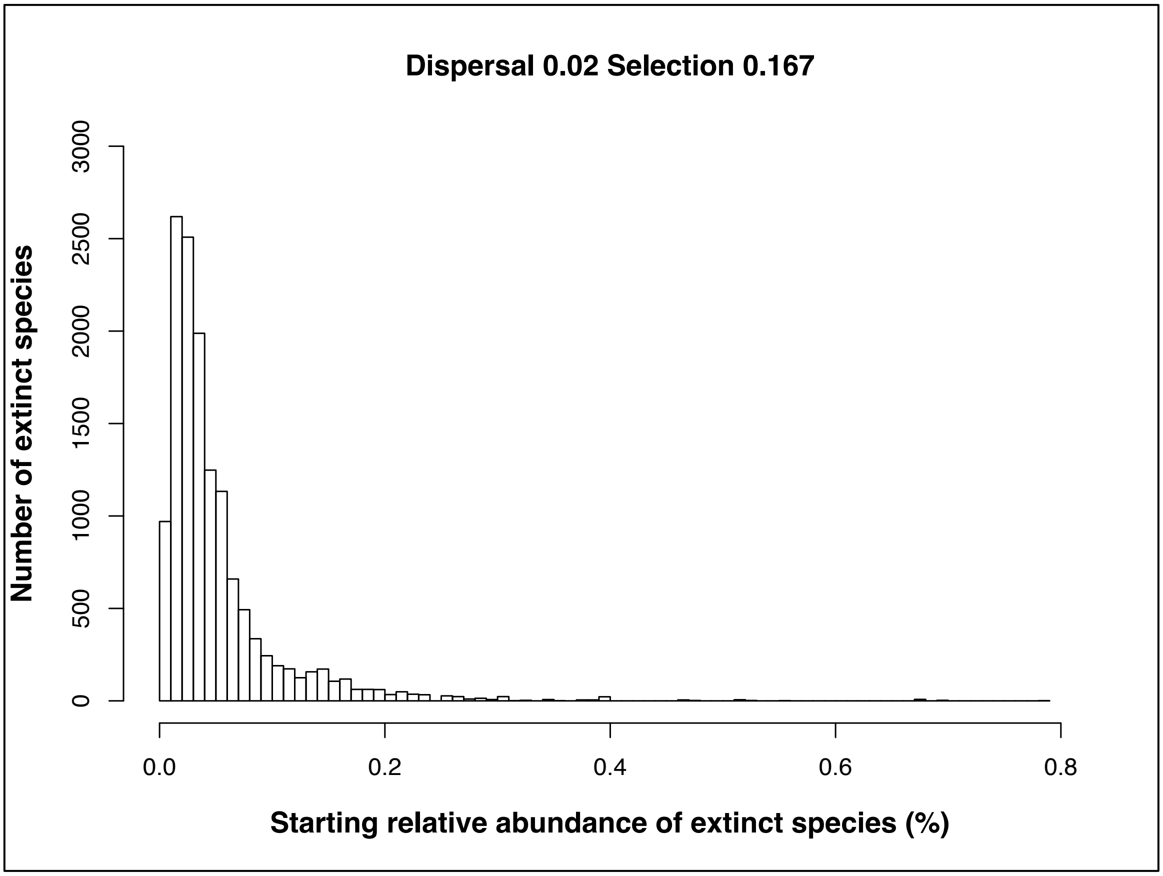

Supplementary Figure S4. A histogram of the starting relative abundances of the extinct species due to drift in the simulation with the lowest dispersal and highest selection. The exact selection and dispersal parameters are shown at the plot title. Note that the histogram shows the total extinct species due to drift in the whole metacommunity rather than the average extinct species per community as the heatmap in Fig. 3B. The distribution is skewed to the right, justifying the use of the median, rather than the mean, to describe the central tendency of the distribution.

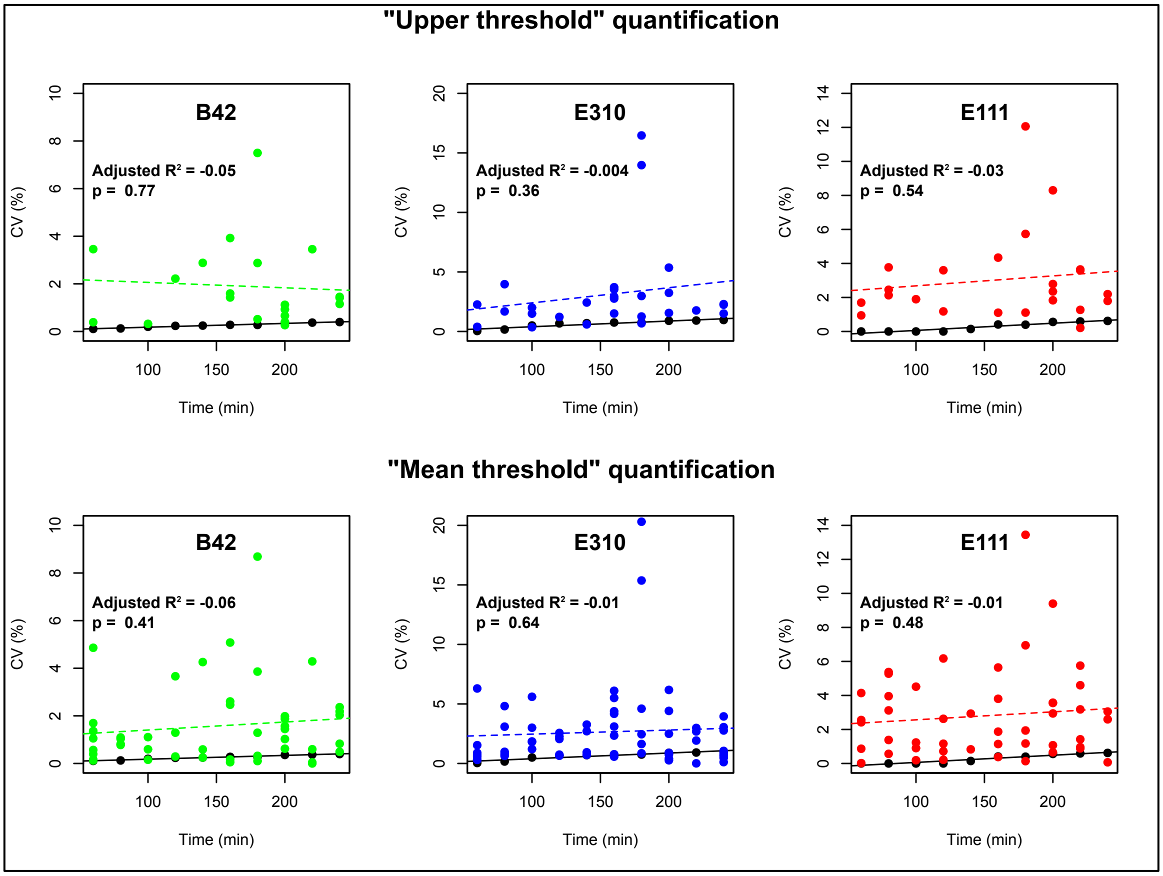

Supplementary Figure S5. The expected deviance from "noise" due to temperature variability in time (black dots and solid lines) and the respective recorded deviance (for both quantification thresholds – colored dots and dashed lines) for each strain. None of the recorded trends are significant and the statistics of the linear correlations are given within each plot. The linear functions of the expected deviance due to temperature variability are as follows: $y = 0.0015x + 0.0196$, $y = 0.0048x - 0.083$ and $y = 0.004x - 0.362$ for B42, E310 and E111, respectively, with $0.84 < R^2 < 0.96$ and $p < 0.001$ for all three.

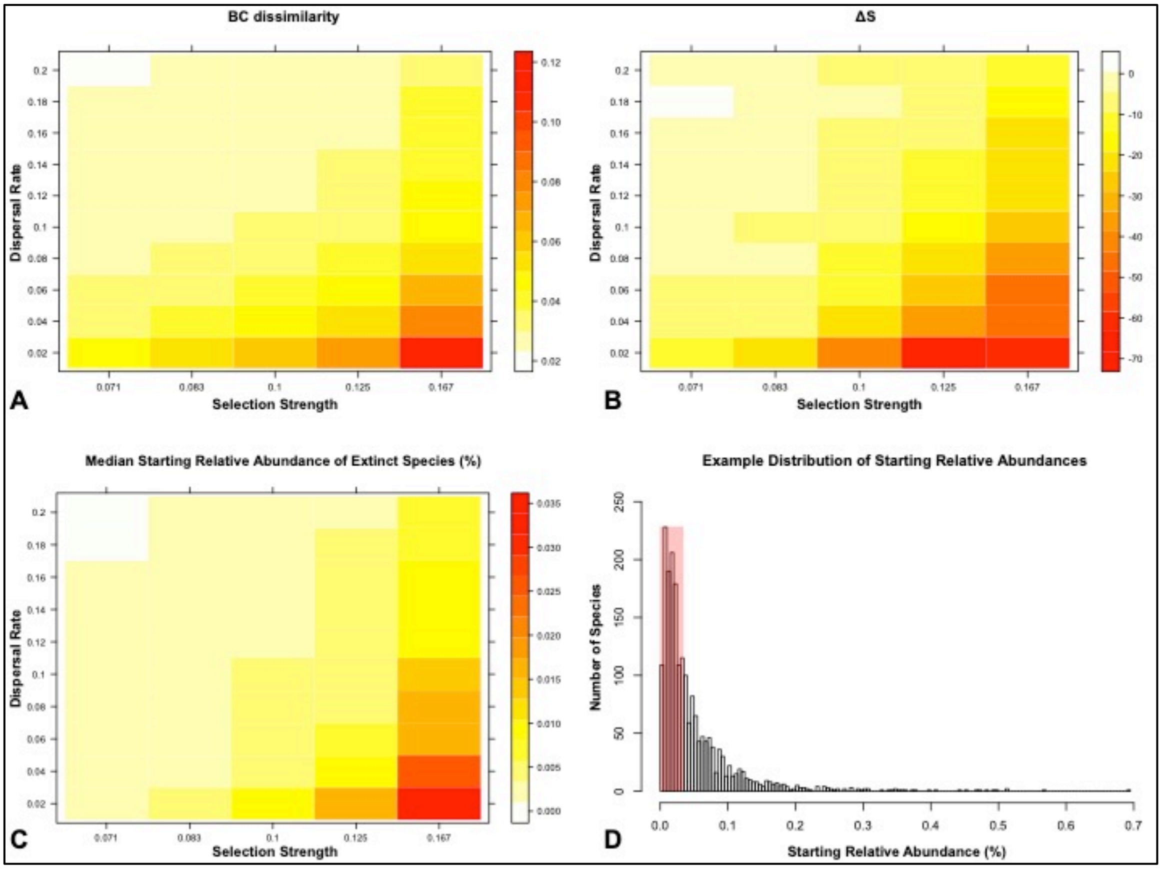

Supplementary Figure S6. The generated β-diversity, the species loss and the starting relative abundances of the extinct species due to drift in the simulations under various dispersal and selection scenarios, with drift simulated as per the "mean threshold" quantification. A. The mean generated β-diversity (Bray-Curtis community dissimilarity – BC) after 1000 generations. **B.** The average difference in the number of species between drift-free and drift-impacted communities (ΔS) after 1000 generations. Comparisons are made with respect to communities growing without drift such that negative values indicate species loss in drift-impacted communities. **C.** The median starting relative abundance (%) of the species that got extinct due to drift after 1000 generations. **D.** An example distribution of the average starting relative abundances in the simulated metacommunities, with the red box representing the range of the median relative abundances of extinct species in the different simulations as per panel C.

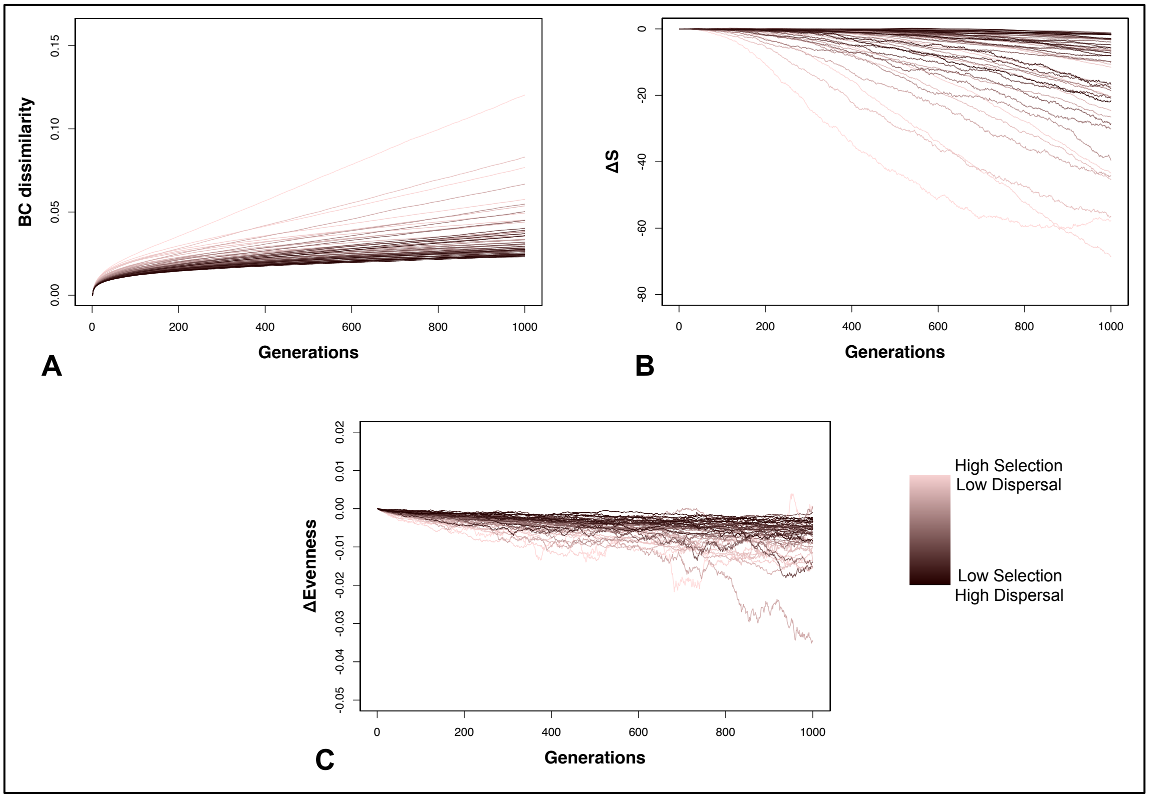

Supplementary Figure S7. The effect of drift (following the "mean threshold" quantification) on the generated β-diversity, on species loss and on community evenness with increasing time in simulations under various selection and dispersal scenarios. A. The generated β-diversity (Bray-Curtis dissimilarity). **B.** The average difference in the number of species between drift-free and drift-impacted communities (ΔS). Negative values indicate species loss in drift-impacted communities. **C.** The difference in Pielou's evenness (ΔEvenness). Negative values indicate lower evenness in drift-impacted communities. The strength of selection and the rate of dispersal change as per the legend on the bottom right.

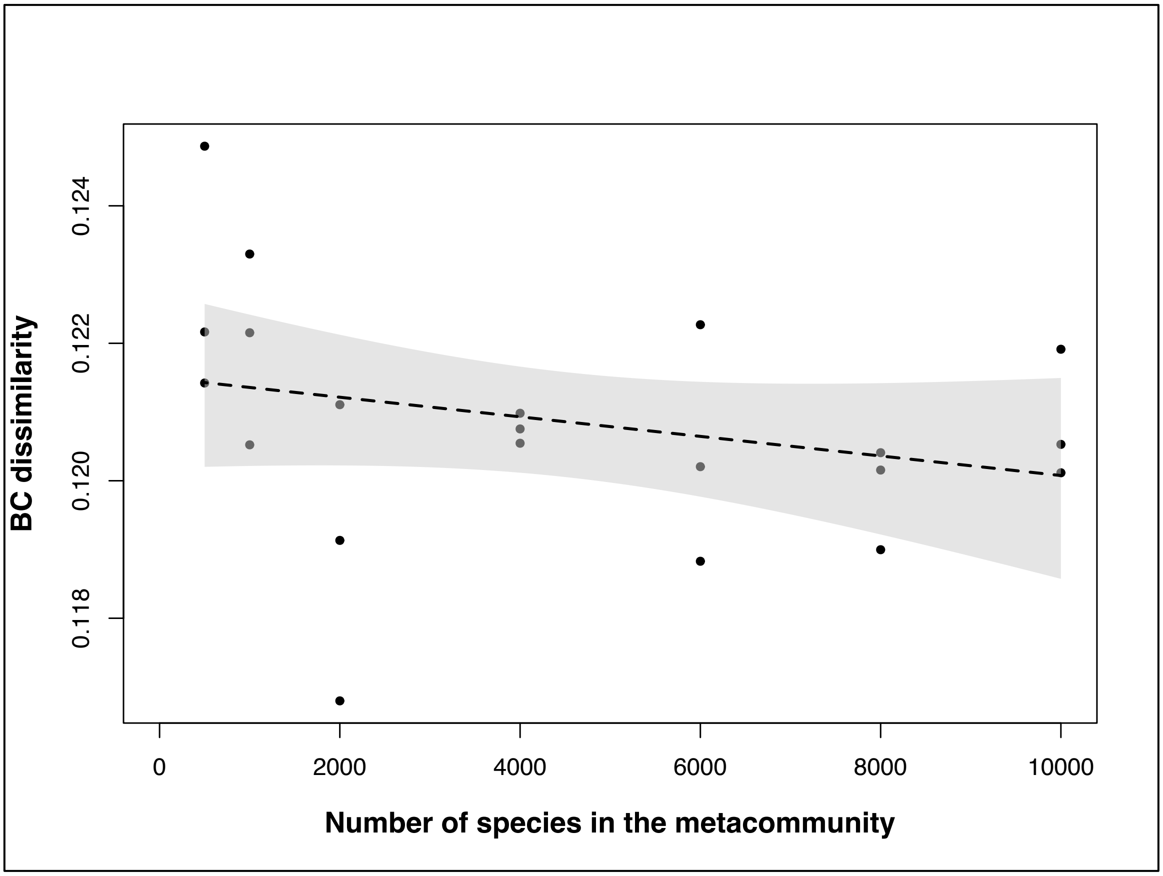

Supplementary Figure S8. The generated β-diversity by drift (Bray-Curtis dissimilarity – BC), following the "mean threshold" drift quantification, (y-axis) as a function of the number of species in the metacommunity (x-axis). Black dots correspond to the values from different simulations (i.e., for metacommunities of 500, 1000, 2000, 4000, 6000, 8000 and 10000 species, n=3 for each level). The line corresponds to the mean and the shaded grey area to the 99.5% confidence intervals of the fitted, non-significant, linear model (adjusted R^2 = 0.035, p = 0.204).

Supplementary Tables

Supplementary Table S1. The fit of the different tested models concerning the “noise” as a function of the total cell density of a given sample. For the model parameters in columns 3-5, the fitted value ± the standard error is reported. ***stands for $p < 0.001$, n/a: not applicable

Model	equation	a	b	c	Residual standard error	AIC
**Exponential**	$f(x) = a \times e^{bx} + c$	51.02 ± 24.95	-1.61 ± 0.40***	2.2 ± 0.52***	2.629	938.21
**Power**	$f(x) = a \times x^{b} + c$	11.76 ± 1.22***	-1.88 ± 0.61**	1.16 ± 1.12	2.651	934.59
**Michaelis-Menten**	$f(x) = a \times x/(b + x)$	1.93 ± 0.16***	-1.04 ± 0.03***	n/a	2.535	942.42

Supplementary Table S2. The estimated parameters and the fit of the models to the distribution of the magnitude of drift following quantification based on the "upper threshold". For columns 2 and 3, the exact terms of the model-specific parameters are mentioned in parentheses and the values are given ± the standard error in the estimation of each parameter.

Distribution	1st parameter	2nd parameter	Log-likelihood	AIC	BIC
**log-normal**	0.625 ± 0.106 (mean)	0.889 ± 0.075 (standard deviation)	-134.832	273.664	278.161
**gamma**	1.439 ± 0.221 (shape)	0.524 ± 0.096 (rate)	-138.173	280.3454	284.8424
**normal**	2.747 ± 0.346 (mean)	2.897 ± 0.245 (standard deviation)	-173.790	351.580	356.078
**weibull**	1.141 ± 0.097 (shape)	2.899 ± 0.322 (scale)	-139.623	283.246	287.743
**exponential**	0.364 ± 0.044 (rate)	n/a	-140.7331	283.4662	285.7147
**cauchy**	1.804 ± 0.165 (location)	0.863 ± 0.137 (scale)	-148.1113	300.222	304.720

Supplementary Table S3. The estimated parameters and the fit of the models to the distribution of the magnitude of drift following the "mean threshold" quantification. For columns 2 and 3, the exact terms of the model-specific parameters are mentioned in parentheses and the values are given ± the standard error in the estimation of each parameter.

Distribution	1st parameter	2nd parameter	Log-likelihood	AIC	BIC
**log-normal**	0.245 ± 0.105 (mean)	1.302 ± 0.074 (standard deviation)	-296.785	597.57	603.643
**gamma**	0.943 ± 0.094 (shape)	0.398 ± 0.052 (rate)	-286.432	576.863	582.937
**normal**	2.366 ± 0.221 (mean)	2.744 ± 0.156 (standard deviation)	-373.948	751.896	757.97
**weibull**	0.948 ± 0.058 (shape)	2.305 ± 0.207 (scale)	-286.2	576.4	582.5
**exponential**	0.423 ± 0.034 (rate)	n/a	-286.6	575.214	578.251
**cauchy**	1.2 ± 0.126 (location)	0.926 ± 0.11 (scale)	-340.349	684.7	690.77

Supplementary Table S4. The type III analysis of variance of the linear mixed effects model that we used to test the dependence of drift (i.e., the experimentally acquired z-scores) on the cell density, on the identity of the strain, on the species richness of the community and on the starting cell concentration. The exact formula used for the lmem() function in R was: *z_score ~ S + Strain + log10(Start_conc) + log10(Cells_ul) + (1|time) + (1|Treatment)*

Factor	Sum of squares	Degrees of freedom (numerical)	Degrees of freedom (denominator)	F value	Pr (> F)
**log(cell density)**	9.54	1	47.54	0.6291	0.4316
**Species Richness**	0.68	1	3.73	0.0451	0.843
**Strain**	36.57	2	65.44	1.2056	0.3061
**log(starting cell density)**	31.4	1	73.43	2.07	0.1545

Supplementary Table S5. Summary of the linear model fits regarding the dependence of the generated β-diversity due to drift on the number of species in the metacommunity in simulations at low selection/high dispersal (0.071 and 0.2, respectively) and medium selection/dispersal (0.1 and 0.1, respectively), using both the "upper threshold" and "mean threshold" quantification.

**Selection/Dispersal**	**"Upper threshold"**			**"Mean threshold"**		
	**R^2**	**F**	**p**	**R^2**	**F**	**p**
**0.071/0.2**	-0.024	0.52	0.48	-0.035	0.31	0.58
**0.1/0.1**	-0.019	0.62	0.44	-0.053	3E-04	0.99

Supplementary Text

Details and example of how drift was modeled in the simulations

For our simulations, we modeled drift exactly like it we quantified it experimentally, i.e., as variation in the growth rates of species. In each of our simulations, we first started with two identical metacommunities containing 100 communities each; d_i and nd_i, respectively with i € [1-100]. At every generation, growth rates were assigned in two stages: 1) at the first stage, <u>identical</u> growth rates were assigned to the species in the respective communities in the two metacommunities, e.g., to species in communities d_1 and nd_1, with these growth rates being picked from a normal distribution with a mean of 1 <u>and an sd equal to the strength of selection</u>. 2) at the second stage, the assigned growth rates of the communities in the metacommunity under the presence of drift (d_i) <u>changed further according to the magnitude of drift.</u> For example, let's assume that in the first stage species α in community d_1 and nd_1 was assigned a growth rate of 1.027 and at the second stage drift was modeled as per the "upper threshold" quantification and a value of 2.41 was picked out of the respective log-normal distribution. The latter value is a CV value, because this is what we measured experimentally. This value indicates that drift would cause a variation in the growth rate that could be described with a normal distribution with mean=0 and sd=1.027*2.41/100. Subsequently, we picked "variations" from the latter distributions and we subtracted them from the original growth rates to create the changed growth rates due to drift (picked values were both positive and negative -because the mean was

0- so that final growth rates could both increase and decrease compared to the originals). The final changed growth rates replaced the original ones in the metacommunity under drift (d_i). For the sake of our example above, a probable picked value from the respective "variation" distribution could be -0.0262 so that the resulting changed growth rate that would be assigned to species α in community d_1 would be 1.0532. As a reminder, the respective growth rate of species α in community nd_1 would still be 1.027 at the same generation. From the above it is evident that the difference between the growth rates of two given communities d_i and nd_i does not depend on selection, but depends only on whether simulations modeled drift following the "upper threshold" or the "mean threshold" quantification.

Supplementary References

1. Fodelianakis S, Lorz A, Valenzuela-Cuevas A, Barozzi A, Booth JM, Daffonchio D. Dispersal homogenizes communities via immigration even at low rates in a simplified synthetic bacterial metacommunity. *Nat Commun*. 2019; **10**:1314.
